# Transparent Nanocomposites Comprising Ligand-Exchanged CuInS_2_/ZnS Quantum Dots and UV-Cured Resin for Wavelength Converters

**DOI:** 10.1021/acsomega.2c02922

**Published:** 2022-09-07

**Authors:** Momo Shiraishi, Yoshiki Iso, Tetsuhiko Isobe

**Affiliations:** Department of Applied Chemistry, Faculty of Science and Technology, Keio University, 3-14-1 Hiyoshi, Kohoku-ku, Yokohama 223-8522, Japan

## Abstract

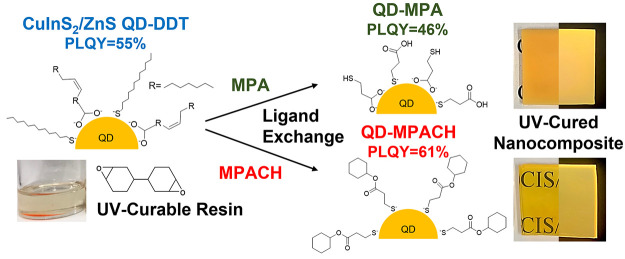

Quantum dots (QDs) dispersed in UV-curable resin are used for patterning in photolithography and inkjet printing. However, low affinity between the main component of UV-curable resins known as celloxide, an alicyclic diepoxy compound, and QD surface ligands with alkyl chains causes significant aggregation of QDs. In this study, the dispersibility of core/shell CuInS_2_/ZnS QDs with adsorbed 1-dodecanethiol and oleic acid in celloxide was improved using the ligand exchange method to prepare transparent fluorescent nanocomposites. Cyclohexyl 3-mercaptopropionate (MPACH) and 3-mercaptopropionic acid (MPA) were successfully adsorbed onto the QDs. MPACH-modified QDs (QD-MPACH) were well dispersed in the UV-curable resin, whereas MPA-modified QDs (QD-MPA) exhibited significant aggregation. Nanocomposite plates containing dispersed QDs were prepared by UV irradiation. The QD-MPACH nanocomposite plate was transparent, while the QD-MPA nanocomposite plate was turbid. The homogeneous dispersion of QD-MPACH was attributed to the similarity in the molecular structure between MPACH and celloxide. The photoluminescence (PL) peak of the QD-MPA nanocomposite occurred at a longer wavelength than that of the QD-MPACH nanocomposite. Furthermore, compared with the absolute photoluminescence quantum yield (PLQY) of the as-prepared QDs in toluene (55%), that of the QD-MPA nanocomposite was smaller (46%), and that of the QD-MPACH nanocomposite was higher (61%). An enhanced self-absorption effect was observed for the QD-MPA nanocomposite because of significant light scattering by the aggregates and concentration quenching, resulting in the PL redshift and decreased PLQY. Moreover, the PL intensity of the QD-MPACH nanocomposite was maintained at 98% of the initial value after continuous excitation-light irradiation for 5 h. The high PLQY and photostability of the QD-MPACH nanocomposite are beneficial in practical applications.

## 1 Introduction

**Figure 1:**
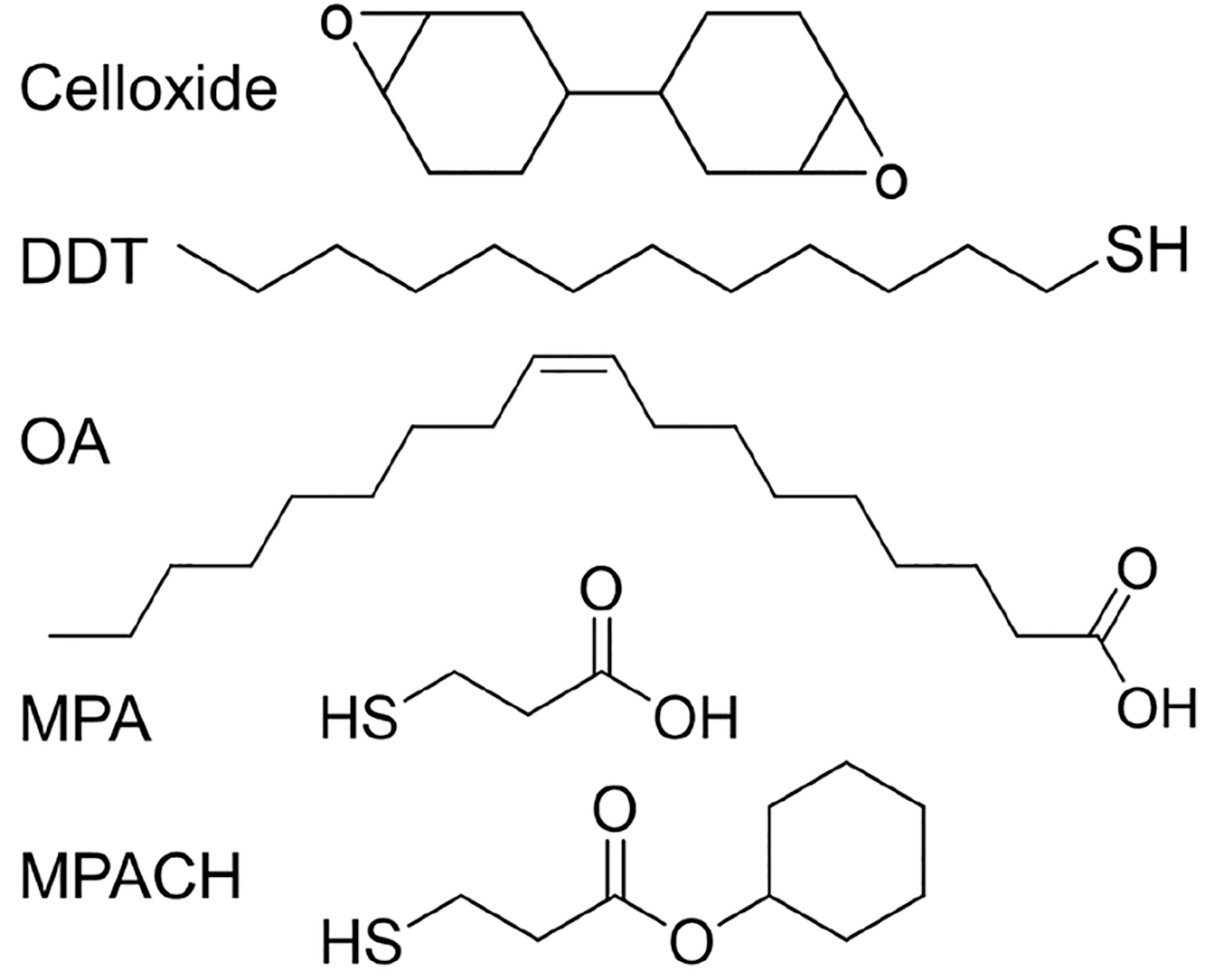
Molecular structures of the compounds used in the preparation of the nanocomposites.

Quantum dots (QDs) produce a single tunable emission peak with high photoluminescence quantum yield (PLQY) through the quantum size effect. Therefore, they are suitable for many applications, including wavelength converters of photovoltaic devices,^1−4^ light-emitting diodes (LEDs),^5−8^ displays,^9−12^ chemo sensors,^13,14^ and bioconjugates.^15,16^ In many device applications, QDs are processed into either films or sheets, such as spectral converting films in solar cells^1−4^ and green- and red-emitting layers in blue LEDs of displays.^11,12^ Patterning, as implemented in photolithography^8,13^ and inkjet printing,^7,11,17^ is a well-known method used to fabricate QD films for optoelectronic devices in which QDs are dispersed in resins cured by UV irradiation. However, as-synthesized QDs with surface ligands containing alkyl chains, such as 1-dodecanethiol (DDT) and oleic acid (OA), cannot be dispersed in UV-curable resins based on celloxide, which is an alicyclic diepoxy compound (see Figure S1). This is attributed to the low affinity between the alkyl groups of the surface ligands on the QDs and the molecular groups of celloxide, as shown in Figure 1. The homogeneous dispersion of QDs in the cured component of the ink is essential to produce high-quality films and sheets. Ligand exchange is a well-known surface modification technique for QDs.^18−20^ Ligand exchange is readily performed by injecting the desired molecule directly into the QD dispersion during the synthesis process.^21−23^ Ligand exchange occurs during the aging process of QDs in which surface ligands adsorb and desorb repeatedly.^24^

In this work, the surface of the lipophilic QDs were modified by ligand exchange with appropriate molecules to improve their dispersibility in celloxide to prepare nanocomposite plates using the fluorescent QDs and the colorless UV-curable resin. Core/shell CuInS_2_ (CIS)/ZnS QDs with adsorbed DDT and OA were prepared as the model material because of their lipophilic surface and high PLQY over 50%.^2^ The CIS/ZnS QDs can also be synthesized via aqueous routes, whereas their PLQYs are much lower.^25^ There are many reports regarding fluorescent QD nanocomposites using acrylic resins prepared by UV-curing technique.^26−28^ On the other hand, to the best of our knowledge, celloxide, which is an alicyclic epoxy compound, has not been investigated as a matrix for fluorescent QD nanocomposites yet. The ligand molecule must have (i) a structure similar to that of celloxide and (ii) functional groups, such as thiol or carboxyl, that are easily adsorbed onto the QD surface.^24,29^ Although our preliminary experiments showed that ligand exchange with cyclohexanethiol improved the dispersibility, the odor was very strong and difficult to handle.^30^ Therefore, cyclohexyl 3-mercaptopropionate (MPACH), which has a similar structure and weaker odor, was considered as a more appropriate surface ligand. This molecule has a six-membered ring, like celloxide, and a thiol group (Figure 1), satisfying the above two conditions. For comparison, 3-mercaptopropionic acid (MPA), which does not have a six-membered ring, was also used for surface modification. The dispersibility of ligand-exchanged QDs in UV-cured resin and the PL properties of the UV-cured nanocomposites were evaluated.

## 2 Experimental Section

### 2.1 Materials

Zinc(II) acetate dihydrate (99.999%), copper(I) iodide (99.999%), and indium(III) acetate (99.99%) were purchased from Sigma–Aldrich. OA (>85.0%), DDT (>95.0%), 1-octadecene (ODE; > 90.0%), and MPA (>98.0%) were purchased from Tokyo Chemical Industry. MPACH (>90.0%) was purchased from FUJIFILM Wako Pure Chemical. Toluene (>99.5%), ethanol (>99.5%), chloroform (99.0%), and hexane (96.0%) were purchased from Kanto Chemical. The used celloxide, an alicyclic epoxy compound, was celloxide 8000 manufactured by TOKYO OHKA KOGYO Co., Ltd. The UV-curable resin composed of celloxide 8000 and a photoacid generator at 2 wt % was also provided by the same company. Toluene, ethanol, chloroform, and hexane were dehydrated over molecular sieves (3A 1/8, FUJIFILM Wako Pure Chemical) prior to use.

### 2.2 QD Preparation

**Figure 2:**
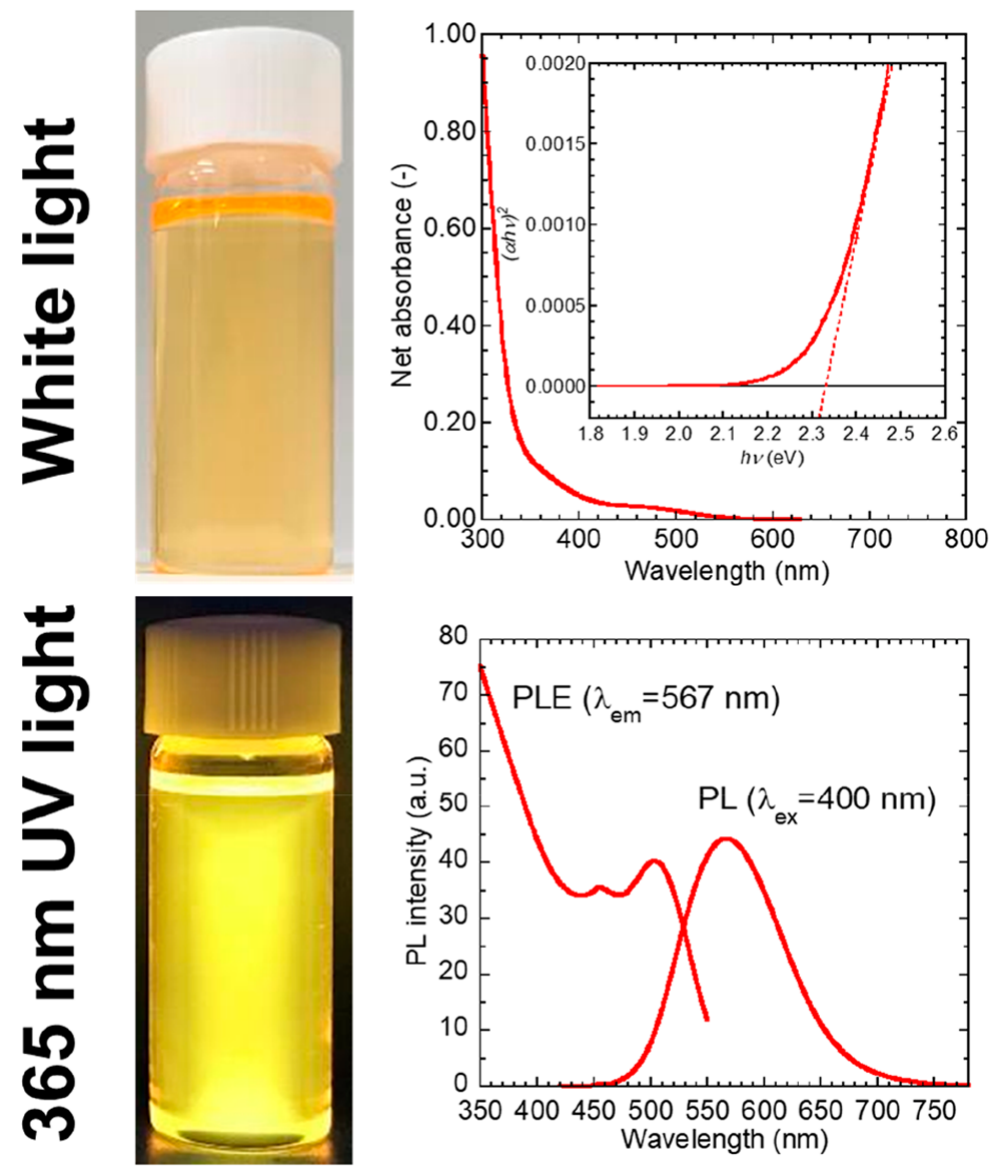
Photographs under white light and 365 nm UV light, UV–vis absorption spectrum and corresponding Tauc plot (inset), and PL and PLE spectra of QD-DDT dispersed in toluene. The net absorbance was calculated by subtracting the absorbance of pure toluene from that of the dispersion.

CIS/ZnS QDs were prepared as described in our previous work.^2^ A mixture of zinc(II) acetate dehydrate (4.00 mmol), oleic acid (1.5 mL), DDT (1.0 mL), and ODE (4.0 mL) was heated at 190 °C for 5 min. The mixture was then bubbled with Ar for 30 min to yield a ZnS shell stock solution. DDT (5.0 mL) was placed in a four-necked flask and bubbled with Ar gas at 450 mL min^–1^ for 30 min. Copper(I) iodide (0.125 mmol) and indium(III) acetate (0.500 mmol) were then added to the four-necked flask. The nominal molar ratio of Cu^+^ to In^3+^ (Cu/In) was adjusted to 1/4. The ZnS shell stock solution was placed in a pressure-equalizing dropping funnel connected to the four-necked flask. The system was degassed at 100 °C for 30 min and purged with Ar gas under stirring. The temperature was increased to 230 °C, and the mixture was maintained at this temperature for 5 min to prepare CIS QDs. The ZnS shell stock solution was then dropped into the resulting dispersion at a rate of 1 mL min^–1^. The temperature was increased to 250 °C, and the mixture was then aged for 50 min. Furthermore, another ZnS shell stock solution was again injected in the same way. The mixture was maintained at this temperature for 60 min to grow the ZnS shell sufficiently. This dispersion was used for ligand exchange. To prepare CIS/ZnS QDs without ligand exchange (QDs-DDT), toluene (7.5 mL) and ethanol (15.0 mL) were added after the dispersion was cooled to room temperature. The aggregated QDs were collected by centrifugation using a rotor (10 cm in radius) spinning at 8000 rpm (∼7000 × g) for 10 min. The obtained precipitate was redispersed into toluene (5.0 mL) under ultrasonication. After the addition of ethanol (15.0 mL), the precipitate was collected by centrifugation for 15 min. This cycle of washing and centrifugation was performed twice. The resulting precipitate was redispersed in toluene to prepare a dispersion of QD-DDT (Figure 2). A powder sample was obtained by drying the precipitate in a vacuum desiccator for 20 h.

### 2.3 Ligand Exchange and Fabrication of Nanocomposite by UV Curing

To prepare MPA-modified QDs (QD-MPA) or MPACH-modified QDs (QD-MPACH), we injected 6 mL of MPA or MPACH into the dispersion after the double-shelled QDs were prepared. After aging at 250 °C for 15 min, the resulting dispersion was cooled to room temperature. Then, toluene (5.0 mL) and ethanol (15.0 mL) were added to the dispersion, and the aggregated QDs were collected by centrifugation using a rotor (10 cm in radius) spinning at 8000 rpm (∼7000 × g) for 10 min. The collected QDs were redispersed into toluene (5.0 mL) under ultrasonication. After adding ethanol (10.0 mL), centrifugation at ∼7000 × g was performed again. The collected precipitate was redispersed in toluene (5.0 mL) and aggregated by adding ethanol (7.5 mL) for further washing. Precipitates of QD-MPA and QD-MPACH were collected by centrifugation at ∼7000 × g. Powder samples were prepared by vacuum drying for 20 h.

Precipitates of QD-MPA and QD-MPACH (0.10 or 0.25 g) were added to pure celloxide or the UV-curable resin (5.0 g) under ultrasonication over 30 min. The UV-curable resins containing QDs were poured into a silicone mold (20 mm × 20 mm × 2 mmt) and cured under a 365 nm UV lamp (UVL-56, Analytik Jena) over 30 min to prepare plate samples at QDs concentrations of 2 or 5 wt %. A blank plate sample without QDs was also prepared in the same way.

### 2.4 Characterization

QD morphologies were observed with a transmission electron microscope (TEM; Tecnai G2, FEI). The TEM sample was prepared by drying a drop of QD dispersion in toluene at 1 mg mL^–1^ on a high-resolution carbon-reinforced copper grid (HRC-C10, Oken Shoji) in an electric desiccator overnight. Powder X-ray diffraction (XRD) profiles were obtained using an X-ray diffractometer (RINT-2200, Rigaku) equipped with a Cu Kα radiation source and a monochromator. X-ray fluorescence (XRF) spectra were measured using a wavelength-dispersive sequential X-ray spectrometer (ZSXmini II, Rigaku). Calibration curves obtained from Cu, In, and Zn standard solutions were used to determine the elemental compositions of the samples from the peak intensities in the recorded XRF spectra. Samples for XRF measurements were prepared by dropping 12 M hydrochloric acid solution (0.20 mL) to dissolve the QD powder sample (6 mg) on a filter paper, followed by drying. Ultraviolet–visible (UV–vis) absorption spectra of liquid samples were measured using a UV–vis optical absorption spectrometer (V-570, JASCO). Tauc plots were constructed in accordance with eq 1 to determine the bandgap energy (*E*_g_) of the QDs:^31^
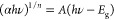
1where α is the absorbance, *h* is the Planck constant, ν is the frequency, and *A* is a constant. We used 0.5 as the value of *n* because CIS is a well-known direct transition-type semiconductor.^32−35^ Transmission spectra of the plate samples were measured with the same apparatus equipped with an integrating sphere (JASCO, ISN-470). Photoluminescence (PL) spectra, PL excitation (PLE) spectra, and changes in the PL intensity under continuous excitation-light irradiation were recorded using a fluorescent spectrometer (FP-6500, JASCO). Absolute PLQYs were measured with a quantum efficiency measurement system (QE-2000-311C, Otsuka Electronics).

## 3 Results and Discussion

### 3.1 Characterization of the As-Prepared CIS/ZnS QDs

The TEM image of as-prepared QD-DDT is shown in Figure S2. Particles were not clearly observed because of electron scattering by residual organic molecules. The particle size of the QDs estimated from the blurring shadow was 2–3 nm, which was consistent with the results (2.6 ± 0.3 mm) of our previous work.^2^ The XRD profile of the powdered QDs is shown in Figure S2. The diffraction peaks of CIS and ZnS were difficult to distinguish. The observed peaks were close to those attributed to the (111), (220), and (311) planes of ZnS, indicating that the amount of ZnS shell was higher than that of the CIS core. This was supported by the measured elemental molar ratio (Cu:In:Zn = 1:4.6:50) using XRF. It should be noted that the Cu/In ratio was close to the preparation ratio, 1:4.

Figure 2 shows the UV–vis absorption and PL spectra of QD-DDT dispersed in toluene. Optical absorption by CIS was observed at wavelengths shorter than ∼550 nm.^36^ The *E*_*g*_ estimated from the Tauc plot converted from the UV–vis absorption spectrum (Figure 2) was 2.33 eV; according to the effective mass approximation,^37^ the diameter of the CIS core was estimated as 2.3 nm. CIS/ZnS QDs exhibited a broad peak corresponding to a defect derived from the Cu^+^ vacancy.^38,39^ The PL peak wavelength and full width at half-maximum (fwhm) were respectively 567 and 103 nm at the excitation wavelength of 400 nm. The absolute PLQY was 55%. The PLE spectrum below ∼550 nm was similar to the UV–vis absorption spectrum.

### 3.2 FT-IR Analysis of QD Surface Ligands

**Figure 3:**
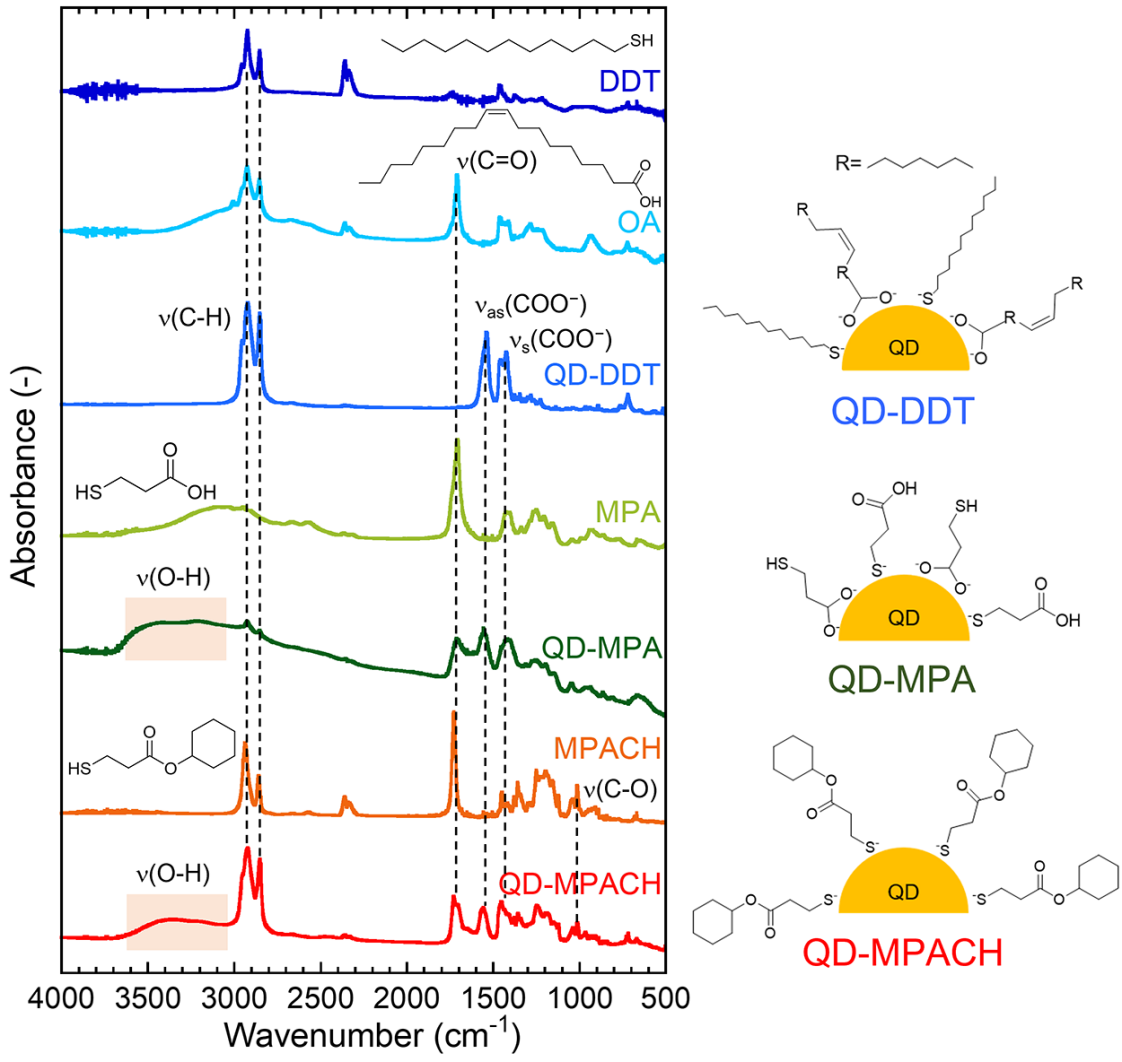
FT-IR spectra of DDT, OA, QD-DDT, MPA, QD-MPA, MPACH, and QD-MPACH. Expected adsorption states of the surface ligands on QDs are also shown schematically.

Figure 3 shows the FT-IR spectra of the prepared QDs and the used ligand molecules. The peak assignments are summarized in Table 1. QD-DDT exhibited strong ν(C–H) peaks attributed to the long alkyl chains of DDT and OA, although the intensity of these peaks decreased for QD-MPA, indicating adsorption of MPA, with shorter alkyl chains, by ligand exchange. Peaks of ν_as_(COO^–^) and ν_s_(COO^–^) were observed for QD-DDT and QD-MPA, revealing the adsorption of the deprotonated carboxyl groups of OA and MPA to the QD surface metals. The ν(C=O) peak appeared for QD-MPA, whereas it was not observed for QD-DDT. MPA was adsorbed onto the QDs via both the carboxyl and thiol groups. Therefore, the carboxyl group of MPA was not deprotonated, as shown in Figure 3. The peaks of ν(O–H) and ν(C–O) were also assigned to the carboxyl group. The peaks of ν(C=O) and ν(C–O) for QD-MPACH were attributed to the ester bond. It should be noted that the peaks of ν_as_(COO^–^) and ν_s_(COO^–^) was also observed, although MPACH does not have carboxyl groups. This result indicated that OA residues existed in QD-MPACH even after ligand exchange, or carboxyl groups were produced from the hydrolysis of the ester bond of MPACH.

**Table 1:** Peak Assignments for the FT-IR Spectra^24,40,41^

wavenumber (cm^–1^)	assignment
3600–3000	ν(O–H)
2930	ν(C–H)
2860	ν(C–H)
1710	ν(C = O)
1550	ν_as_(COO^–^)
1420	ν_s_(COO^–^)
1000	ν(C–O)

### 3.3 Dispersibility of the QDs and Optical Properties of UV-Cured Nanocomposite Plate

**Figure 4:**
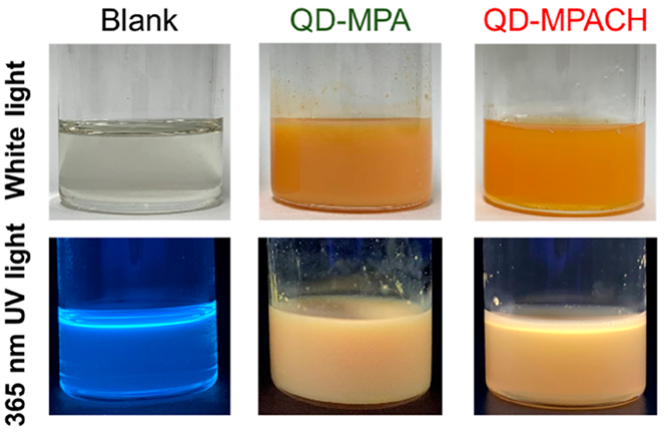
Photographs of the blank UV-curable resin, QD-MPA dispersion, and QD-MPACH dispersion under white light and 365 nm UV light.

QD-MPA and QD-MPACH were dispersed in UV-curable resin at 5 wt %, as shown in Figure 4. QD-MPACH was more dispersed than QD-MPA. Both dispersions showed yellowish white luminescence under 365 nm UV light. The observed PL color was a mixture of yellow from the CIS/ZnS QDs and blue from the UV-curable resin. Pure celloxide did not exhibit visible PL under UV excitation, whereas the UV-curable resin composed of the photoacid generator and celloxide showed blue PL, as shown in Figure S3. Therefore, the blue emission was derived from the photoacid generator. It should be noted that this unnecessary blue emission was negligible when the QDs were irradiated by excitation light of wavelengths longer than ∼450 nm, which was not absorbed by the UV-curable resin, as confirmed from its absorption spectrum.

**Figure 5:**
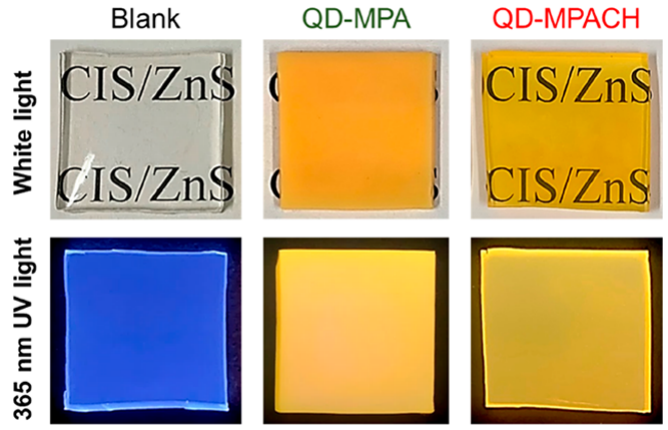
Photographs of the blank UV-cured resin plate and nanocomposite plates of QD-MPA and QD-MPACH at 5 wt % (20 mm × 20 mm × 2 mmt) under white light and 365 nm UV light.

Figure 5 shows photographs of the UV-cured nanocomposite plates. The blank plate was colorless and transparent under white light. The QD-MPA nanocomposite plate was turbid, while the QD-MPACH nanocomposite plate was clear. Under 365 nm UV light, the blank plate showed blue emission, while both QD-dispersed nanocomposite plates showed yellow emission.

**Figure 6:**
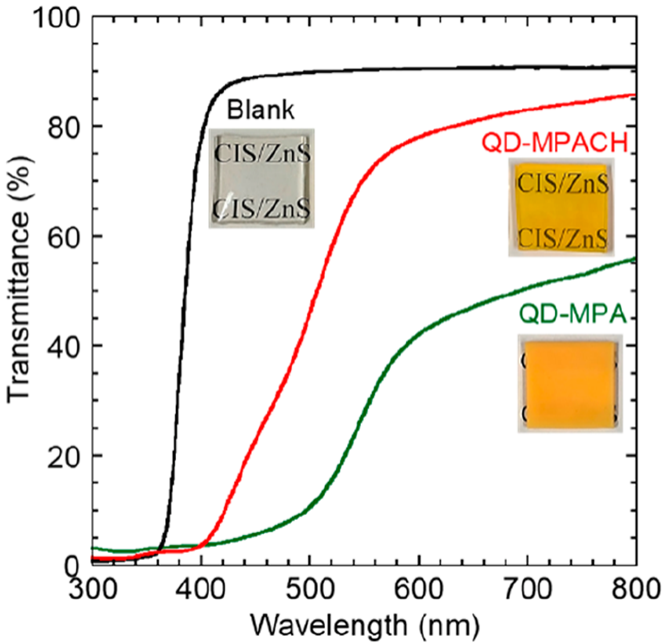
Transmission spectra of the blank UV-cured resin plate and nanocomposite plates of QD-MPA and QD-MPACH at 5 wt %.

The transmission spectra of the plates are shown in Figure 6 (see also corresponding UV–vis absorption spectra in Figure S4). The highest transmittance was observed for the blank plate in the whole wavelength region. The transmittance of the QD-MPACH nanocomposite plate was higher than that of the QD-MPA nanocomposite plate. The prepared QDs did not absorb light of wavelengths longer than ∼550 nm, as shown in Figure 2. Therefore, the decrease in transmittance at 550–800 nm was attributed to light scattering loss. According to the effective medium approximation model, QD dispersion may increase the average refractive index of the plate, increasing the reflectance. Because the QD concentration was small (5 wt %), this effect on the refractive index was negligible. Therefore, by suppressing QD aggregation through improvement of the affinity between the QD surface and celloxide, the transparency of the QD-MPACH nanocomposite plate was higher than that of the QD-MPA nanocomposite plate. This excellent affinity would be attributed to structural similarity between MPACH and celloxide, which have the alicyclic structure as displayed in Figure 1.

**Figure 7:**
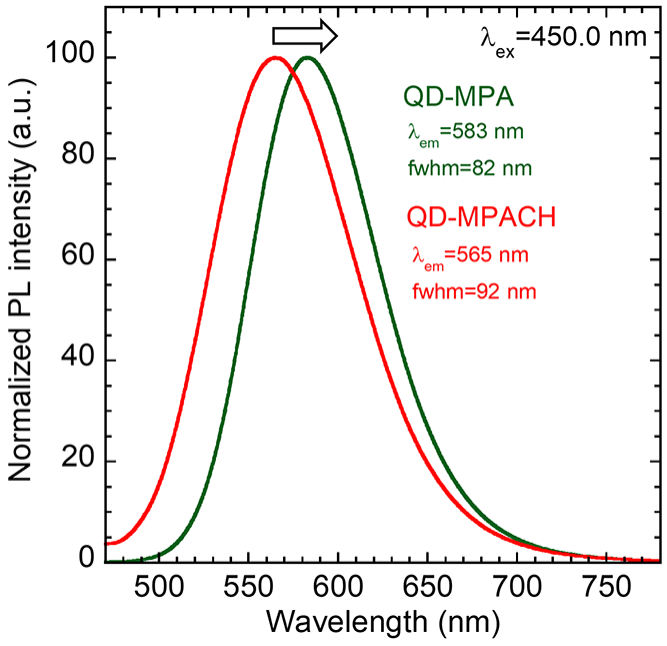
PL spectra for nanocomposite plates of QD-MPA and QD-MPACH at 5 wt %.

**Figure 8:**
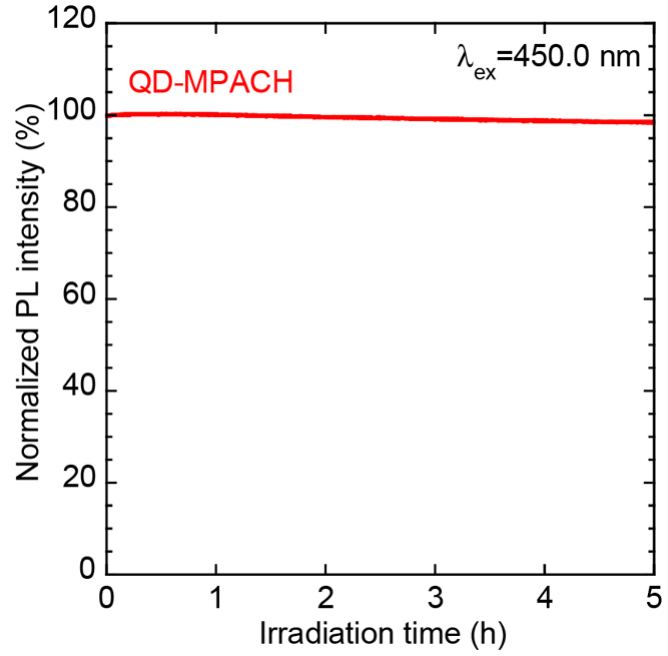
Change in the PL intensity of the QD-MPACH nanocomposite plate at 5 wt % under continuous irradiation at 450.0 nm for 5 h.

The normalized PL spectra are shown in Figure 7. In this study, excitation at 450 nm was used to avoid blue emission from the photoacid generator in the UV-cured resin. The PL peak of the QD-MPACH nanocomposite plate appeared at 565 nm, which was very close to 567 nm of the QD-DDT in toluene (Figure 2), revealing good dispersion of QD-MPACH in the resin. On the other hand, the PL peak of the QD-MPA nanocomposite plate appeared at 583 nm. The obvious redshift occurred because significant aggregation of QD-MPA led to strong self-absorption,^42^ while good dispersion of QD-MPACH resulted in weak self-absorption. To confirm the influence of self-absorption, we also fabricated nanocomposite plates with QDs at a lower concentration (2 wt %) (Figure S5). The PL spectra of the QD-MPA dispersed plates (Figure S6) showed that a decrease in the QD concentration resulted in a blueshift of the PL peak by 10 nm and an increased fwhm of 6 nm, indicating that the influence of self-absorption was suppressed. In contrast, the QD-MPACH dispersed plate exhibited a PL blueshift of 6 nm with an increased fwhm of 1 nm. These changes were smaller than those of the QD-MPA dispersed plate. Light scattering from QD-MPACH was small because of its higher dispersibility in the resin, resulting in smaller changes in the PL spectrum when the QD concentration was reduced. The absolute PLQY of the 5 wt % QD-MPA dispersed plate (46%) was smaller than that of the as-prepared QDs in toluene (55%). This could be attributed to concentration quenching by strong aggregation and increased surface defects due to the ligand exchange process. In contrast, the absolute PLQY of the 5 wt % QD-MPACH dispersed plate was 61%. Therefore, the density of adsorbed surface ligands (MPACH) was likely high, leading to further passivation of the QD surface. This nanocomposite was continuously irradiated with excitation light for 5 h. As shown in Figure 8, 98% of the initial PL intensity was maintained after 5 h, revealing that the QD-MPACH plates were highly photostable. In our previous work, the powdered CIS/ZnS QDs measured under the same conditions exhibited a decrease in the PL intensity to 88% after 5 h.^22^ Because CIS/ZnS QDs are degraded by photo-oxidation with oxygen, the UV-cured resin may protect the dispersed QDs from oxygen in air, thus improving the photostability.

## 4 Conclusions

In this work, MPACH and MPA were adsorbed onto CIS/ZnS QDs through the ligand exchange method and the modified QDs were dispersed in UV-curable resin. QD-MPA showed drastic aggregation, whereas QD-MPACH was well dispersed because the molecular structures of MPACH and celloxide were similar. Nanocomposite plates were prepared by irradiating the UV-curable resin containing dispersed QDs with UV light. The PL peak of the turbid QD-MPA nanocomposite was located at a longer wavelength than that of the transparent QD-MPACH nanocomposite. Furthermore, compared with the absolute PLQY of the as-prepared QDs in toluene (55%), that of the QD-MPA nanocomposite was smaller (46%), and that of the QD-MPACH nanocomposite was higher (61%). These results could be explained by the enhanced self-absorption effect in the QD-MPA nanocomposite arising from significant light scattering by the aggregates and concentration quenching. The PL intensity of the QD-MPACH nanocomposite was maintained at 98% of the initial value even after continuous excitation-light irradiation for 5 h. High PLQYs and photostability facilitate the applications of these nanocomposites, for example, as wavelength converters of photovoltaic devices, LEDs, and displays.

## Supporting Information Available

Schematic illustration of CIS/ZnS QD with adsorbed DDT and OA and photographs of the QDs in the UV-curable resin (Figure S1); XRD profile and TEM image of as-prepared CIS/ZnS QDs (Figure S2); photographs, UV–vis absorption spectra, and PL spectra of pure celloxide, the UV-curable resin liquid (Figure S3); UV–vis absorption spectra of the blank UV-cured resin plate and nanocomposite plates of QD-MPA and QD-MPACH at 5 wt % (Figure S4); photographs of the blank plate and nanocomposite plates of QD-MPA and QD-MPACH at 2 and 5 wt % (Figure S5); normalized PL spectra of the nanocomposite plates of QD-MPA and QD-MPACH at 2 and 5 wt % (Figure S6) (PDF)
