# Supplementary material for: Transparent Nanocomposites Comprising Ligand-Exchanged CuInS2/ZnS Quantum Dots and UV-Cured Resin for Wavelength Converters
Source: ACS Omega. 2022 Sep 7;7(37):33039–45. doi: 10.1021/acsomega.2c02922 (PMC9494423; doi:10.1021/acsomega.2c02922)
Supplement: Supplementary file 1 — ao2c02922_si_001.pdf [file ao2c02922_si_001.pdf]

Supporting Information

ACS Omega

Transparent Nanocomposites Comprising Ligand-Exchanged $CuInS_2$/ZnS Quantum Dots and UV-Cured Resin for Wavelength Converters

Momo Shiraishi, Yoshiki Iso,* Tetsuhiko Isobe,*

Department of Applied Chemistry, Faculty of Science and Technology, Keio University, 3-14-1 Hiyoshi, Kohoku-ku, Yokohama 223-8522, Japan

*Corresponding Authors:

Yoshiki Iso – E-mail: iso@applc.keio.ac.jp; Tel.: +81 45 566 1558; Fax: +81 45 566 1551

Tetsuhiko Isobe – E-mail: isobe@applc.keio.ac.jp; Tel.: +81 45 566 1554; Fax: +81 45 566 1551

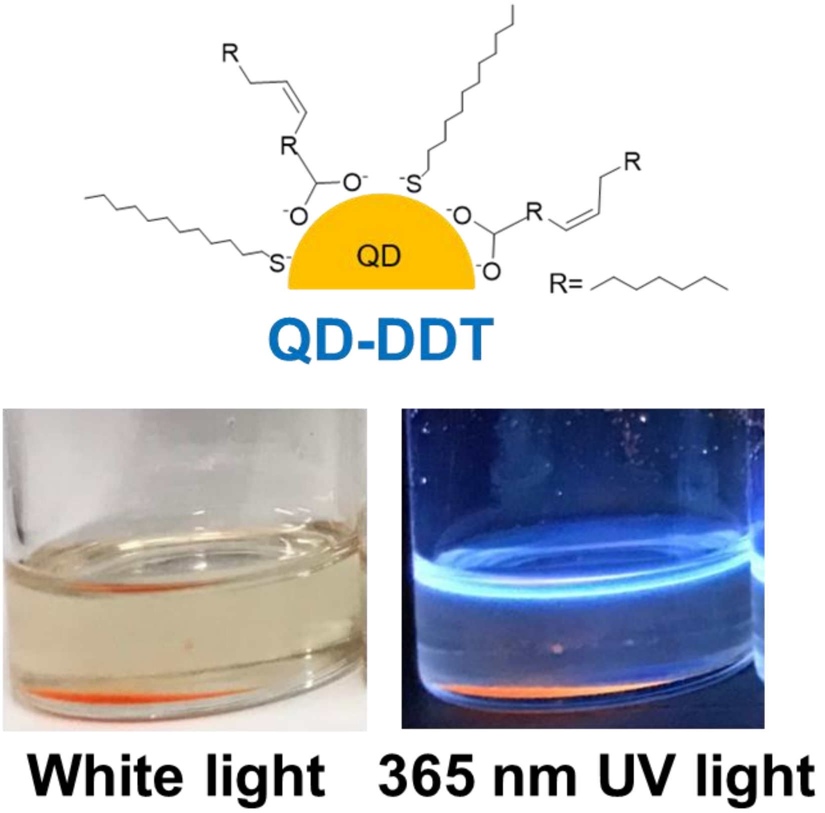

Figure S1. Schematic illustration of CIS/ZnS QDs with adsorbed DDT and OA and photographs of the QDs in the UV-curable resin under white light and 365-nm UV light.

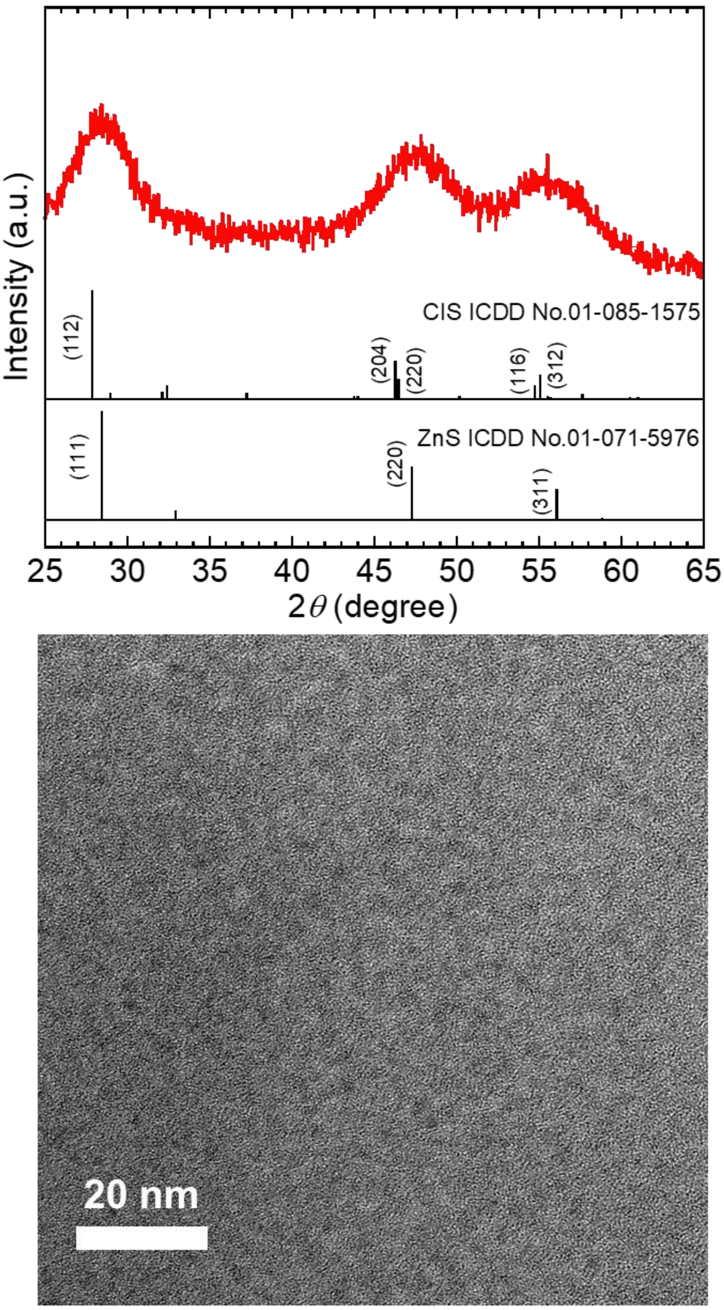

Figure S2. XRD profile and TEM image of as-prepared CIS/ZnS QDs.

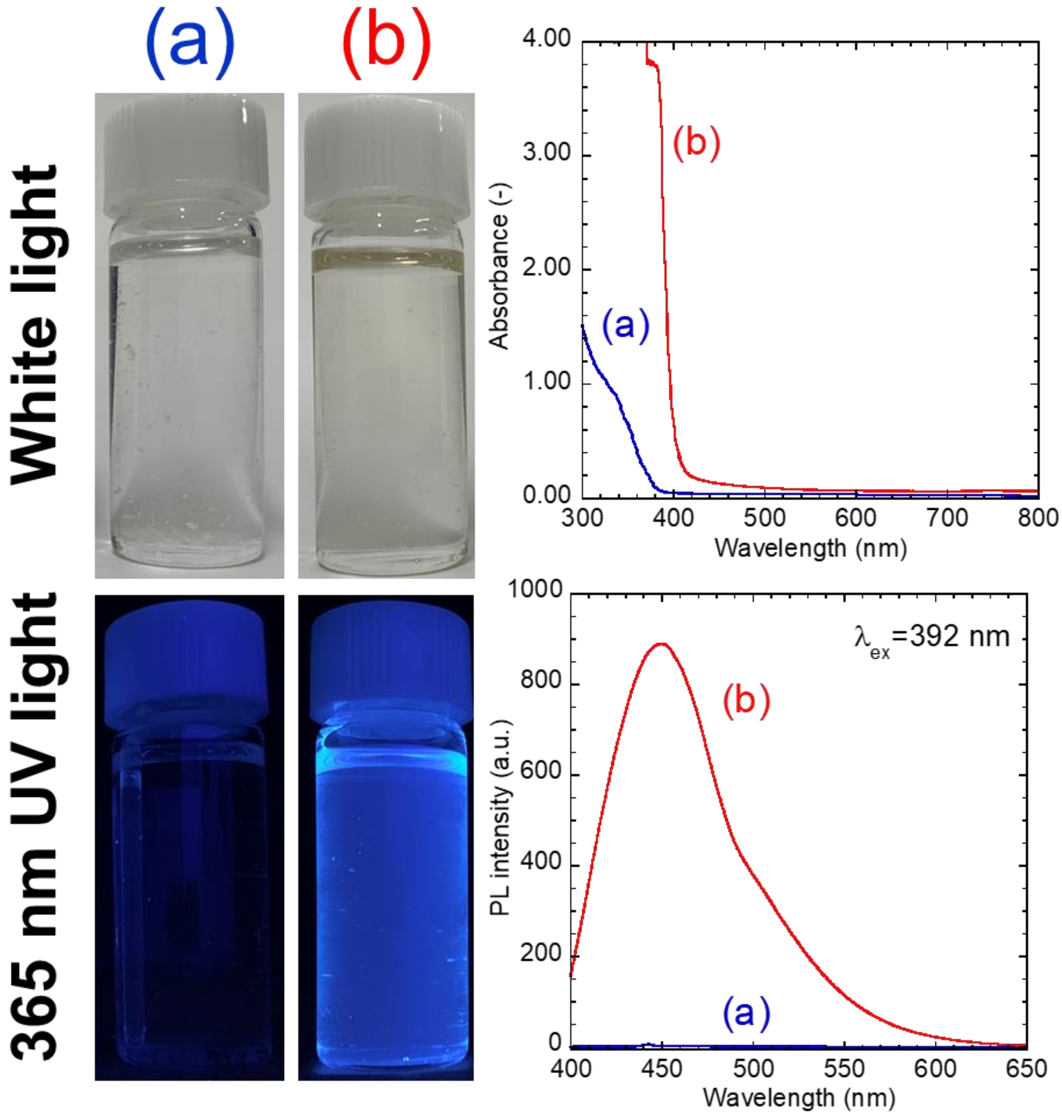

Figure S3. Photographs, UV–vis absorption spectra, and PL spectra of (a) pure celloxide and (b) the UV-curable resin liquid composed of celloxide and the photoacid generator at 2 wt%.

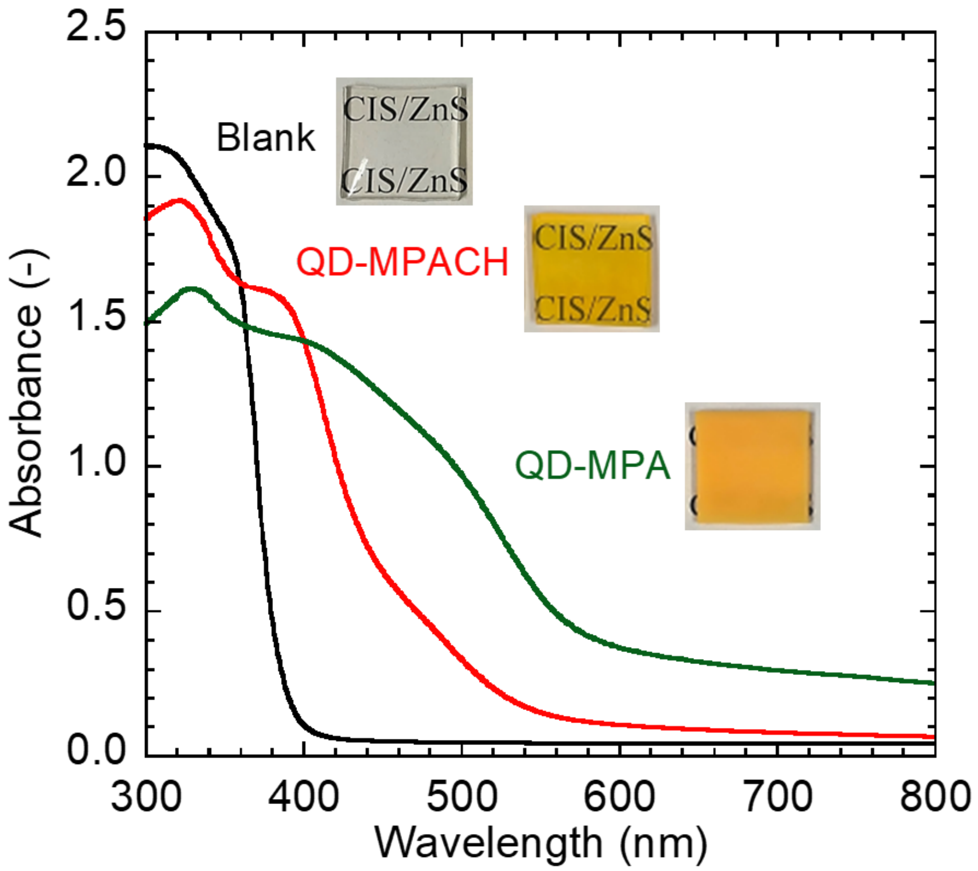

Figure S4. UV-vis absorption spectra of the blank UV-cured resin plate and nanocomposite plates of QD-MPA and QD-MPACH at 5 wt%.

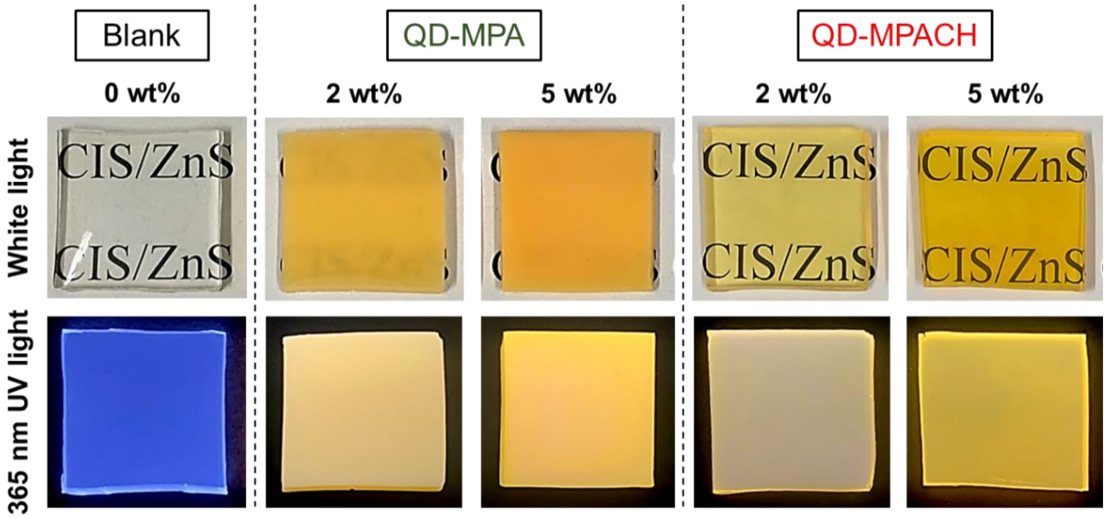

Figure S5. Photographs of the blank plate and nanocomposite plates of QD-MPA and QD-MPACH prepared at 2 wt% and 5 wt%.

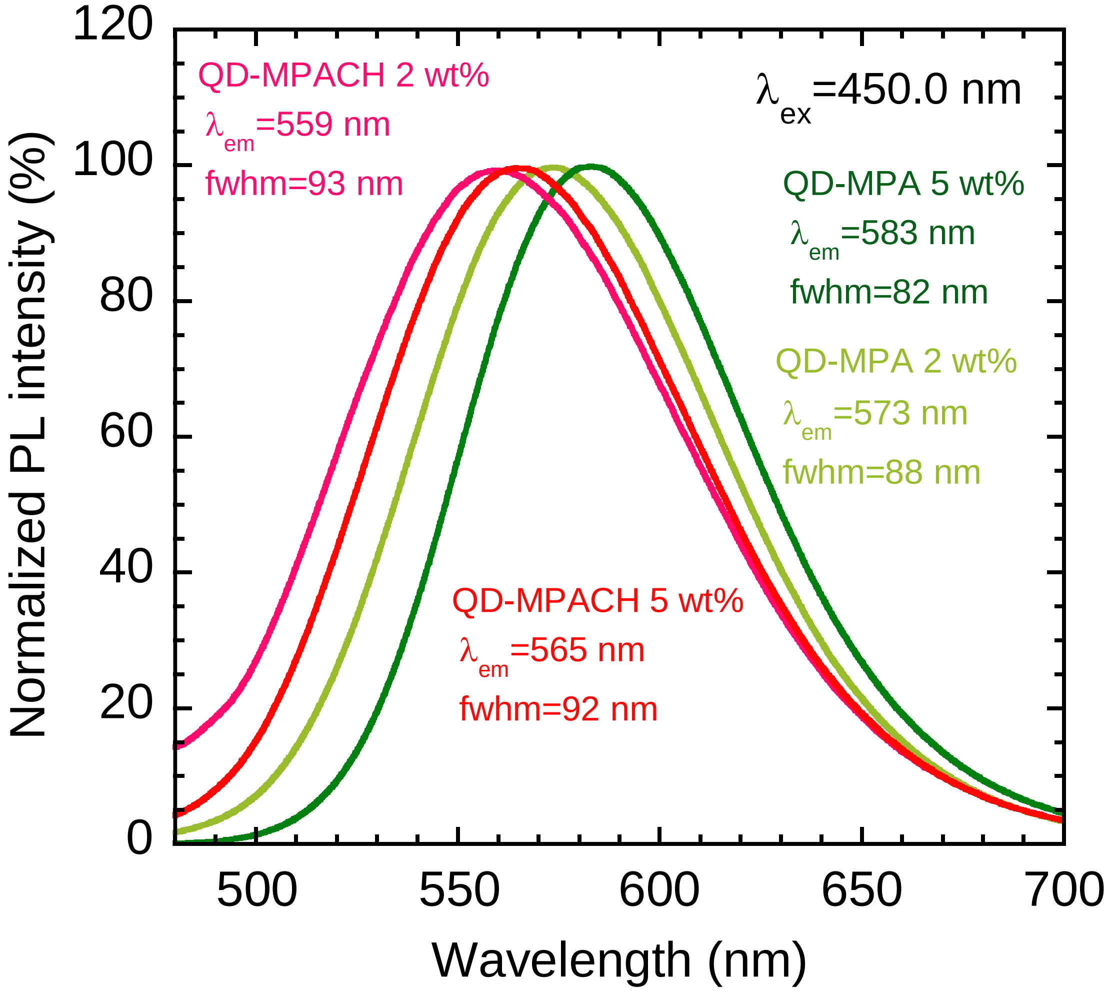

Figure S6. Normalized PL spectra of the nanocomposite plates of QD-MPA and QD-MPACH prepared at 2 wt% and 5 wt%.
